# Physiological adaptations during weaning from veno-venous extracorporeal membrane oxygenation

**DOI:** 10.1186/s40635-023-00493-8

**Published:** 2023-02-10

**Authors:** Patrick Duncan Collins, Lorenzo Giosa, Valentina Camarda, Luigi Camporota

**Affiliations:** 1 Department of Critical Care Medicine, Guy’s and St. Thomas’ National Health Service Foundation Trust, London, UK; 2 Centre for Human and Applied Physiological Sciences, School of Basic and Medical Biosciences, King’s College London, London, UK

**Keywords:** Extracorporeal membrane oxygenation (ECMO), Weaning, Acute respiratory distress syndrome (ARDS), Respiratory drive, Patient self-inflicted lung injury (P-SILI)

## Abstract

Veno-venous extracorporeal membrane oxygenation (V–V ECMO) has an established evidence base in acute respiratory distress syndrome (ARDS) and has seen exponential growth in its use over the past decades. However, there is a paucity of evidence regarding the approach to weaning, with variation of practice and outcomes between centres. Preconditions for weaning, management of patients’ sedation and mechanical ventilation during this phase, criteria defining success or failure, and the optimal duration of a trial prior to decannulation are all debated subjects. Moreover, there is no prospective evidence demonstrating the superiority of weaning the sweep gas flow (SGF), the extracorporeal blood flow (ECBF) or the fraction of oxygen of the SGF (FdO2), thereby a broad inter-centre variability exists in this regard. Accordingly, the aim of this review is to discuss the required physiological basis to interpret different weaning approaches: first, we will outline the physiological changes in blood gases which should be expected from manipulations of ECBF, SGF and FdO2. Subsequently, we will describe the resulting adaptation of patients’ control of breathing, with special reference to the effects of weaning on respiratory effort. Finally, we will discuss pertinent elements of the monitoring and mechanical ventilation of passive and spontaneously breathing patients during a weaning trial. Indeed, to avoid lung injury, invasive monitoring is often required in patients making spontaneous effort, as pressures measured at the airway may not reflect the degree of lung strain. In the absence of evidence, our approach to weaning is driven largely by an understanding of physiology.

## Take home message

Successful weaning from veno-venous ECMO requires understanding and consideration of the complex physiological interactions between the native and the extracorporeal lung. We describe our understanding of the physiological adaptations of the patient as extracorporeal support is weaned and outline the implications for clinical practice.

## Introduction

Veno-venous extracorporeal membrane oxygenation (V–V ECMO) is a technique that can support gas exchange and enable a reduction in the mechanical power applied to the injured lung in patients with acute severe and refractory, but potentially reversible, respiratory failure.

The increasing emphasis on reducing ergotrauma, the technological improvements in the extracorporeal devices, and the viral pandemics (i.e. H_1_N_1_ influenza and SARS-CoV 2) have resulted in an exponential growth in ECMO utilization over the last decade [1]. The possible applications of V–V ECMO are wide, including severe Acute Respiratory Distress Syndrome (ARDS), near fatal asthma, severe air leak syndromes, interstitial lung disease or as a bridge to lung transplantation [2–6].

In contrast to the abundance of data on indications, complications and prognostic factors for ECMO survival [1–3], consensus guidelines on weaning [6] are based on limited direct evidence on the criteria to initiate a weaning trial, how to monitor patients undergoing weaning and how to adjust mechanical ventilation to optimally support patients during this phase.

In Table 1 [3, 6, 8–20], we summarize the different approaches to weaning of V–V ECMO reported in the literature. Most centres wean the sweep gas flow (SGF) to zero but variable practice regarding manipulation of extracorporeal blood flow (ECBF) or the fraction of oxygen of the SGF (FdO2) is apparent between centres. Furthermore, the monitoring criteria and duration of a weaning trial are extremely variable, with few centres reporting objective assessment of respiratory drive and effort.

**Table 1:** Varied approaches to weaning in the literature

Source	Preconditions for weaning	Preferred ventilation	Targeted parameters during weaning				Measured effort/drive	Monitoring criteria for successful trial
			ECBF (L/min)	FdO_2_	SGF (L/min)	Duration (h)		
Sen et al. 2016 [8]	PEEP 5–10, peak pressure 20–25, TV 6 ml/kg, RR ~ 15, PaO_2_ 50–80, radiological improvement	Controlled or spontaneous	2	0.21	0	Unspecified	–	Clinician Discretion
Reeb et al. 2017 [9]	Sats > 88% (PaO_2_ > 60), FiO_2_ ≤ 0.6, PEEP ≤ 15, RR ≤ 35	Not specified	Unspecified	1.0	0	4	–	Stable ABG
Combes et al. 2018 [3]	Clinical + radiological improvement	Controlled	Unspecified	1.0	0	≥ 1	–	PaO_2_ > 70 mmHg on FiO_2_ < 0.6 plateau pressure < 30. No acute cor pulmonale
Broman et al. 2018* [10]	FiO_2_ 0.35–0.55, minimal V’CO_2ML_ with 5% CO_2_ added to SGF < 2L/min	Controlled or spontaneous	Unspecified	1.0	0	≥ 2–12	–	Clinician discretion
Broman et al. 2018^†^ [10]	FiO_2_ < 0.45, PEEP < 10, peak pressure < 27	Not specified	1.5	1.0	0	0.5–1	–	Stable ABG and absence of dyspnoea
Broman et al. 2018^‡^ [10]	Clinical + radiological improvement	Spontaneous	2.5–3	1.0	0	Unspecified	–	Absence of dyspnoea
Grant et al. 2018 [11]	Sats > 90%, FiO_2_ ≤ 0.5, PEEP ≤ 10, plateau pressure ≤ _2_5, TV ≤ 6-8ml/kg	Controlled	3–4	0.21	≤ 1L	Unspecified	–	Stable ABG, maintain preconditions
Seiler et al. 2018 [12]	Clinician discretion	Controlled	2	1.0	0	1	–	Stable ABG
Chaves et al. 2019 [13]	FiO_2_ ≤ 0.6, PEEP ≤ 15, peak pressure ≤ 30, TV ≤ 6ml/kg, RR ≤ 35 and radiological improvement	Spontaneous	Unspecified	1.0	0	6	–	Clinical stability, normal pH and PaO_2_
Vasques et al. 2019 [14]	Sats > 88% on FiO_2_ 0.6, PaO_2_ > 225 on Cilley test, V’CO_2NL_ > 50% of total, TV ≤ 6–8 ml/kg,	Spontaneous	Unspecified	0.21	0	Unspecified	Yes	P 0.1 > − 10, RR ≤ 35, ratio of V’CO_2_NL to minute ventilation > 80% of baseline, absence of distress
Li et al. 2020 [15]	Clinical + radiological improvement	Controlled	2.5	1.0	0	24–48	–	RR ≤ 20, P:F ratio > 150, Murray Index 2–3, PaCO_2_ ≤ 50, temperature < 38c
Gannon et al. 2021 [16]	SGF ≤ 3, Sats ≥ 88% (PaO_2_ ≥ 60) with FiO_2_ ≤ 0.6, PEEP ≤ 15. RR ≤ 35, HR < 120, systolic BP ≥ 180 or < 90, pH ≥ 7.35	Controlled or spontaneous	< 3	0.5	0	0.5	–	Maintain non-ECMO preconditions, ≤ 20% change in HR
Tonna et al. (ELSO guideline), 202 [6]	PaO_2_ ≥ 70 FiO_2_ ≤ 0.6, PEEP ≤ 10, plateau ≤ 28, TV ≤ 6ml/kg, RR ≤ 28, improved CXR	Controlled or spontaneous	1–1.5**	0.21	0	≥ 2–3	–	Normocapnia, PaO_2_ > 70, no respiratory distress
Teijeiro et al. 2021 [17]	No air leak, No NMB > 24 h, FiO_2_ ≤ 0.6 Sats > 88% PaO_2_ > 60 peak pressure ≤ 20, TV ≤ 9ml/kg, haemodynamically stable, SGF < 5, ECBF < 5,	Spontaneous	< 5	1.0	0	2–24	–	Respiratory distress, TV > 9ml/kg, Sats < 88% (or requiring FiO_2_ > 0.6, PEEP ≥ 20) pH < 7.25, haemodynamic instability, agitation or drowsiness
Belliato et al. 2021 [18]	Clinical and radiological improvement, PEEP ≤ 10–15, haemodynamic stability	Controlled or spontaneous	Unspecified	1.0	0	6–12	–	Clinician discretion
Al-Fares et al. 2021 [19]	Clinician discretion	Controlled or spontaneous	> 3	1.0	0	Unspecified	Yes	Clinician discretion
Lazarri et al. 2022 [20]	∆Pes ≤ 15, RR ≤ 30, pH > 7.25, PaCO_2_ ≤ 60, PaO_2_ > 70 with FiO_2_ ≤ 0.6	Controlled or spontaneous	Unspecified	1.0	0	Unspecified	Yes	Maintain preconditions

ECBF: extracorporeal blood flow, FdO_2_: fraction of oxygen of the Sweep Gas Flow (SGF), PEEP: positive end expiratory pressure, TV: tidal volume (per kilogram of predicted body weight), RR: respiratory rate, ABG: arterial blood gas, PaO_2_: partial pressure of arterial oxygen, PaCO_2_: the partial pressure of arterial carbon dioxide, V’CO_2ML_: carbon dioxide cleared by the membrane lung, V’CO_2NL_: carbon dioxide cleared by the native lung, ∆Pes: the change in oesophageal pressure, P:F ratio: ratio of PaO_2_ to FiO_2_. All airway pressures measured in centimetres of water. All non-airway pressures (including partial pressures) measured in millimetres of mercury. Broman et al. reported an approach from the Karolinska institute*, Regensburg Hospital^†^ and San Raffaele Hospital^‡^. **A reduction in ECBF is considered optional within the guidelines

This variation in practice may contribute to inter-centre heterogeneity in V–V ECMO outcome [1]. Identifying strategies which can accelerate the safe liberation of patients from ECMO are essential to reduce length of stay and risk of complications, as well as ensuring equity of access at times of strain on healthcare resources [7].

A sound understanding of the physiological interactions between the extracorporeal circuit, the patient and the ventilator is required to guide physicians throughout the process of weaning. Accordingly, this review aims to describe a possible physiological approach to weaning from V–V ECMO.

## Physiology of weaning from V–V ECMO

### The extracorporeal circuit

#### V’O_2ML_, V’CO_2ML_ and the effects of weaning

**Fig. 1:**
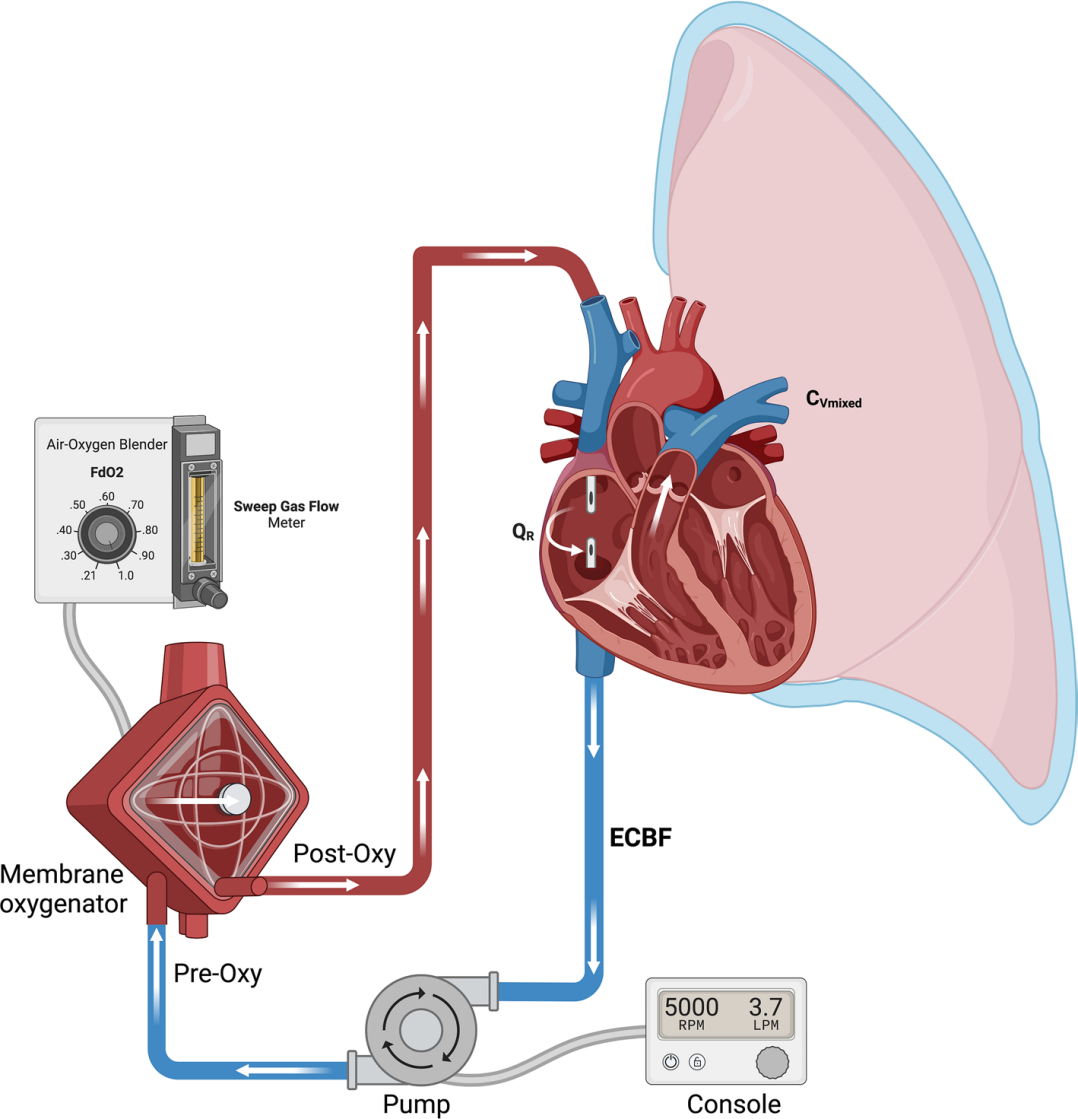
The anatomy and physiology of the extracorporeal circuit, depicted in a femoral–jugular configuration. Blood is drained from the central venous system (C_V_) via a cannula and centrifugal pump which generates extracorporeal blood flow (ECBF). Pre-oxygenator blood is a mixture of central venous (C_v_) and recirculating (Q_R_) blood. It is pumped across hollow fibres within the membrane oxygenator across which there is sweep gas flow (SGF). Post-oxygenator blood passes through the return lumen where it becomes mixed with the C_V_ blood in the right ventricle and pulmonary arteries to form the mixed venous blood (C_Vmixed_) before being distributed through the native pulmonary circulation. Mixed central venous bloods oxygen content (C_Vmixed_O_2_) will be determined by: the central venous oxygen content (C_vO2_), the post-oxygenator blood’s oxygen content (C_post-oxy O2_), the extracorporeal blood flow (ECBF), recirculation flow (Q_R_) and overall cardiac output (Qt) according to the formula: C_Vmixed_O_2_·Qt = [C_v_O_2_·(Qt-ECBF + Qr)] + [C_post-oxy_O_2_·(ECBF-QR)]. Although the ECBF contributes to the calculation of the overall CO_2_ clearance of the membrane lung [V’CO_2ML_ = (C_post-oxy_CO_2_ − C_post-oxy_CO_2_)·ECBF·25)], during the usual V–V ECMO ECBF levels (> 2.5 L) the primary determinant of V’CO_2_ML is the SGF rate which generates the gradient for CO_2_ diffusion and resulting difference in pre- and post-oxygenator CO_2_ content

The extracorporeal circuit is depicted in Fig. 1. Table 2 summarizes the effects of weaning different ECMO parameters on V’O_2ML_ and V’CO_2ML_. There are three main settings which can be manipulated during the weaning or trial off V–V ECMO [21]:

**Table 2:** Titratable ECMO parameters during weaning and their effects

Parameter which can be weaned	Relationship with V’O_2ML_	Relationship with V’CO_2ML_	Downsides of weaning in isolation
ECBF	Linear relationship if Q_R_ is minimal	Linear relationship between 0 and 0.5 L/min Logarithmic relationship between 0.5 and 1 L/min Minimal change > 1 L/min Influenced by membrane lung surface and SGF	Low ECBF flows may increase circuit thrombosis Changes in ECBF will also affect Q_R_
SGF	Minimal change until almost zero	Decrease V’CO_2ML_	When SGF is turned to zero V’O_2ML_ ceases suddenly but hypoxic pulmonary vasoconstriction takes minutes to react
FdO_2_	Decrease V’O_2ML_	No effect	Weaning may alter the respiratory quotient and reduce alveolar oxygen

ECBF: extracorporeal blood flow

SGF: sweep gas flow

FdO_2_: fraction of oxygen of the SGF

Q_R_: blood flow directly back into the ECMO circuit which has already passed through the membrane lung

#### Effects of reducing ECBF

The ECBF rate has differing effects on the oxygen delivery (V’O_2ML_) and CO_2_ clearance of the membrane lung (V’CO_2ML_). With a well-functioning circuit, nearly all haemoglobin passing through the membrane lung will become 100% saturated even at very low SGF rates. Consequently, if the FdO_2_ is unchanged, the ECBF is the main titratable variable which can affect the V’O_2ML_. However, the nature of the relationship between ECBF and the V’O_2ML_ is affected by the amount of recirculated blood flow (Q_R_):

1. Recirculation occurs when arterialized blood returned to the venous system after passing through the membrane lung is aspirated straight back into the circuit (see Fig. 1), lowering the gradient between the pre- and post-membrane blood oxygen content and therefore the V’O_2ML_. *Effective* ECBF, equal to total ECBF minus Q_R,_ is linearly related to V’O_2ML_ [21] but Q_R_ cannot be easily quantified at the bedside.
2. The proportion of Q_R_ may be higher at higher ECBF. If a given decrease in ECBF during weaning disproportionately reduces Q_R_ then the change in V’O_2ML_ may not be as anticipated. For example, if the Q_R_ reduces from 1 L to 250 mL when the ECBF is weaned by 25% from 4 to 3 L/min, then the effective ECBF has only changed from 3 L to 2.75 L. Conversely, when Q_R_ is minimal decreasing the total ECBF will decrease the V’O_2ML_ linearly [21].

The effect of weaning the ECBF upon the systemic oxygenation will depend on the cardiac output and venous admixture: as the proportion of the total cardiac output captured into the ECBF falls, the mixed venous oxygen content will decrease, and the final effect upon systemic oxygenation will be determined by native lung function.

In contrast to V’O_2ML_, the relationship between ECBF and V’CO_2ML_ is not linear, but follows a natural logarithmic curve with ECBF > 0.5 mL/min, which plateaus at > 1.0 L/min [22, 23]. The impact of the ECBF on V’CO_2ML_ is also affected by the ratio of SGF:ECBF and the surface area of the membrane lung [22]. Accordingly, step-wise decreases in ECBF have minimal independent effect on the V’CO_2ML_ until reaching very low levels- which are generally avoided to prevent circuit thrombosis.

#### Effects of reducing SGF rate without altering the FdO_2_

Nearly complete saturation of haemoglobin can be achieved even with very low SGF rates (< 0.5 L/min) particularly when FdO_2_ is maintained at 1.0 [21]. For this reason, step decreases in SGF do not affect V’O_2ML_ until SGF is almost off. Moreover, even the small amount of SGF can affect ECMO dependency for another reason: V–V ECMO causes a mixed venous ‘hyperoxia’, blunting or abolishing the physiological hypoxic pulmonary vasoconstriction [24]. This results in an increased native lung venous admixture, with lower than expected ventilation to perfusion (Va/Q) ratio [24–26]. Accordingly, when the SGF is turned to zero at the last step of a weaning trial, patients are abruptly totally dependent on the native lungs capacity to transfer oxygen (V’O_2NL_), but the biphasic response of the pulmonary vasculature to hypoxia requires minutes to hours to occur [27]. Delayed hypoxic vasoconstriction increases the effective venous admixture, worsening V/Q matching and potentially leading to hypoxaemia which might be avoided with a slower transition.

In contrast to oxygenation, step-wise decreases in SGF are associated with a progressive reduction in V’CO_2ML_ [22]. SGF drives bulk transfer of CO_2_ out of the artificial membrane and increases the gradient for CO_2_ in the venous blood to diffuse across the membrane. Accordingly, step decreases in SGF, result in a higher CO_2_ in the pulmonary vasculature and greater load to the native lung (V’CO_2NL_), whose exchange capacity will affect PaCO_2_. Several indices have been proposed for a bedside evaluation of the CO_2_ clearance capacity of the natural lung, as summarized in Table 3 [19, 20, 28, 29]. Interestingly, the partial pressure of end tidal to arterial PCO_2_ ratio (P_ET_:PaCO_2_), an index of global gas-exchange efficiency [30], was the best predictor of weaning outcome in a recent study [together with a measure of ventilatory efficiency (ratio of respiratory effort to V’CO_2NL_)] [20].

**Table 3:** Evaluation of CO_2_ clearance of native lung during weaning from V–V ECMO

Parameter	Formula	Downsides/specifics
Ventilatory ratio [28]	(VE∙PaCO_2_)/(PBW∙100∙37.5)	Assumes constant V’CO_2NL_
Enghoff index	(PaCO_2_ -PECO_2_)/PaCO_2_	Evaluates both shunt and dead space
Ratio of end tidal to partial pressure of carbon dioxide	EtCO_2_/PaCO_2_	Evaluates both shunt and dead space [20]
Bohr alveolar dead space	(PACO_2_ -PECO_2_)/PACO_2_	Evaluates pure alveolar dead space but requires analysis of volumetric capnography curve [24]
Ventilatory efficiency	VE/ V’CO_2NL_	No available data during ECMO
Ventilatory efficiency	∆P_eso_/ V’CO_2NL_	Influenced by lung elastance

PBW: predicted body weight

VE: minute ventilation

PECO_2_: mean expired CO_2_ partial pressure

PACO_2_: alveolar CO_2_ partial pressure

When SGF is set to zero, V–V ECMO makes no contribution to gas exchange and, after the restoration of hypoxic pulmonary vasoconstriction, a true assessment of native lung function can occur. A special case in which a sudden reduction in arterial oxygenation is unrelated to a lung function and severe hypoxaemia occurs when there is an intracardiac shunt: in this case, the flow from the return cannula can force blood through the shunt, bypassing the native lung and potentially leading to profound desaturation when SGF falls to zero. This will become evident if ECBF is not concomitantly reduced to < 1 L/min when no compensatory oxygen is added from the extracorporeal circuit [31].

#### Effects of reducing the FdO_2_ prior to reducing the SGF

Gradually reducing the FdO2 leads to a sequential decrease in V’O_2ML_, while V’CO_2ML_ remains unaltered. The progressive decrease in FdO_2_ has the advantage of allowing time to restore hypoxic pulmonary vasoconstriction [21]. This will prevent rapid desaturations and provides a more accurate assessment of the oxygen exchange capacity of the native lung. The effect on systemic oxygenation of a reduction in V’O_2ML_ through step decreases in FdO_2_ will largely depend on the ratio of ECBF to cardiac output and on the venous admixture of the native lung.

However, reductions in FdO_2_ may have counter-intuitive effects on the alveolar oxygen partial pressure. If FdO_2_ is weaned in isolation (i.e. SGF is maintained constant), the contribution of V’O2_ML_ to total V’O2 falls, whilst the proportion of the total metabolically produced CO_2_ cleared by the natural lung remains largely unchanged, or even decreased if metabolic CO_2_ production increases with the hypoxic drive, and the native lung is unable to increase CO_2_. In this case, the CO_2_ removed extracorporeally proportionally increases. This means that the respiratory quotient of the natural lung (RQ_NL_ = V’CO_2NL_: V’O_2NL_) will be reduced [32]. The implications of this can be seen from the alveolar gas equation:

$${\mathrm{PAO}}_{2}={\mathrm{PiO}}_{2}-\frac{{\mathrm{PACO}}_{2}}{{\mathrm{RQ}}_{\mathrm{NL}}},$$

where PAO_2_ and PACO_2_ are the alveolar partial pressures of oxygen and carbon dioxide, respectively, and PiO_2_ is the pressure of inspired oxygen.

The greater the reduction in RQ_NL_ from the V’CO_2ML_, the lower the PAO_2_. In other words, if PaCO_2_ is static, the V’CO_2ML_ entails a relative alveolar hypoventilation. This leads to uptake of O_2_ by the lungs which is not compensated for by bulk gas transfer, resulting in a lower PAO_2_. However, the clinical significance of this effect during weaning from V–V ECMO should be put into context:

1. The extended alveolar equation has a supplementary term (in bold here below) which, accounting for changes in alveolar gas volume during breathing, blunts the deleterious effect of low RQ on PAO_2_, especially if the FiO_2_ from the native lung is high [33, 34]:
  $${\mathrm{PAO}}_{2}={\mathrm{PiO}}_{2}-\frac{{\mathrm{PACO}}_{2}}{{\mathrm{RQ}}_{\mathrm{NL}}}+\mathrm{ Fi}{\mathrm{O}}_{2}\bullet \mathrm{ PAC}{\mathrm{O}}_{2}\bullet \frac{1-{\mathrm{RQ}}_{\mathrm{NL}}}{{\mathrm{RQ}}_{\mathrm{NL}}}.$$
2. The reduction in PAO_2_ at low RQ_NL_ is most clinically relevant during extracorporeal CO_2_ removal (ECCO_2_R) [32], where ECBF is < 1 L/min. However, this will be less evident during weaning of V–V ECMO where the ECBF is much higher: indeed, even at FdO_2_ 0.21, there may still be a substantial V’O_2ML_ [21].

### The patient

The patient’s response to a weaning trial is dictated by the physiology of breathing control and, particularly, by the effects of variations in gas-exchange on the output of the respiratory centres.

#### Physiology of breathing control

**Fig. 2:**
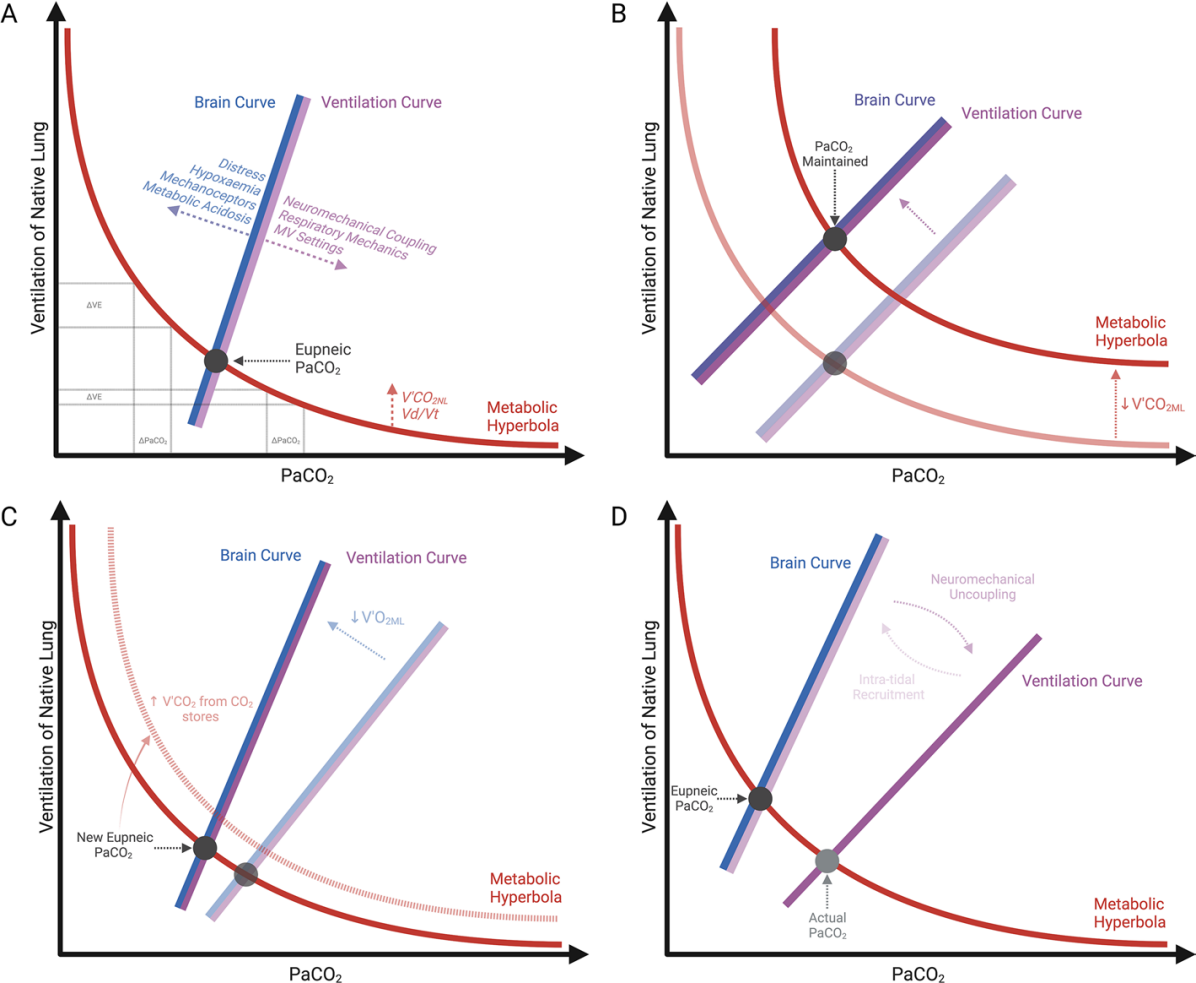
Georgopolous model of breathing control during weaning. **A** Reflects health, were the brain and ventilation curve are synonymous, thereby eupneic and actual PaCO_2_ coincide. Possible contributors to the position of the brain curve, ventilation curve and metabolic hyperbola, and the varying slope of the latter (larger ∆VE required to achieve a given ∆PaCO_2_ when ventilation is higher) are also shown. **B** Represents a possible SGF weaning trial off: as the CO_2_ cleared by the membrane lung (V’CO_2ML_) is reduced the metabolic hyperbola shifts upwards and to the right, while the brain and ventilation curve shift in parallel to the left to maintain the eupneic PaCO_2_. **C** Represents a possible FdO_2_ or ECBF weaning trial off: as the oxygen provided by the membrane lung (V’O_2ML_) is reduced during weaning any hypoxaemia would shifts the position and slope of the brain curve a new eupneic PaCO_2_. This new equilibration point will drive non-metabolic CO_2_ from body stores into the bloodstream possibly shifting the metabolic hyperbola upward and to the right. **D** Depicts possible weaning-induced changes in the relative position of the brain and ventilation curves (maintained synonymous for simplicity in all other Panels): note that any deviation between the two curves induces a difference between the actual and eupneic CO_2_. MV: mechanical ventilation, PaCO_2_: the partial pressure of arterial carbon dioxide, V’CO_2NL_: the total CO_2_ to be cleared by the natural lung, Vd/Vt the dead space fraction of the tidal volume, V’CO_2ML_: the CO_2_ cleared by the membrane lung

A simple, yet effective model describing the control of breathing has been proposed by Georgopoulos et al. [35] and subsequently adopted by others [36]. As depicted in Fig. 2 [35–38], this model describes the interdependence between the arterial partial pressure of CO_2_ (PaCO_2_) and the minute ventilation (VE) by plotting them in the same graph according to three different curves: (1) the *metabolic hyperbola,* describing the relationship between PaCO_2_ and VE at a given V’CO_2NL_ and dead space (Vd/Vt); (2) the CO_2_ sensitivity curve (also called the *brain curve*), describing the change in VE that the respiratory centres desire when PaCO_2_ deviates from its set-point; (3) the *ventilation curve,* depicting the corresponding change in VE that the respiratory system can actually achieve for a given PaCO_2_. In health, the brain and ventilation curves are synonymous.

The intersection between the brain curve and the metabolic hyperbola gives the ‘eupneic’ PaCO_2_, i.e. the PaCO_2_ set-point of the respiratory centres. Conversely, the intersection between the ventilation curve and the metabolic hyperbola gives the actual PaCO_2_ of the patient. Panel A in Fig. 2 describes physiological and pathological determinants of the slopes and positions of these three curves [35, 36].

#### Effects of weaning on respiratory centres output

According to the Georgopoulos model, weaning may affect the respiratory centres’ output through the following mechanisms.

#### Step decreases in SGF may change the position of the metabolic hyperbola

Normally, around 6–7 L/min of VE is sufficient to maintain a PaCO_2_ at 40 mmHg at physiologic VCO_2NL_. During V–V ECMO, VCO_2NL_ decreases and the metabolic hyperbola shifts downward and to the left (i.e. lower VE is required to maintain the same PaCO_2_). Conversely, during weaning, step decreases in SGF rate reduce V’CO_2ML_ and the total metabolic V’CO_2_ increases at higher work of breathing. For both reasons, V’CO_2NL_ is expected to increase, shifting the metabolic hyperbola upward and to the right (see Panel B in Fig. 2).

#### Step decreases in FdO_2_ or ECBF may change the set-point of the brain curve

Normally, the brain is set to maintain a PaCO_2_ around 40 mmHg. However, chemical (PaO_2_ and pH), reflex (lung and chest wall receptors) and cortical (wakefulness, sedation, agitation) inputs can change the set-point to lower or higher values. In ARDS, stimulation of lung mechanoceptors and inflammation contribute to a low PaCO_2_ set-point even in normoxia. For this reason, even with maximal V’CO_2ML_ it is uncommon to induce apnoea during V–V ECMO for ARDS [39]. During weaning, step decreases in V’O_2ML_, may induce hypoxemia, thereby lowering the PaCO_2_ set-point. This will result in the brain curve shifting to the left and increasing its slope (see Panel C in Fig. 2).

#### Changes in breathing pattern may affect the ventilation curve

In health, ventilation satisfies the activity of the respiratory centres, thereby the ventilation and brain curve overlap and the actual PaCO_2_ matches the eupnoeic PaCO_2_. In ARDS, the descending pathway from the brain to the lung is altered. Dissociation between the two curves results in dyspnoea and further increasing the already high respiratory centres output. During weaning, elicited natural lung ventilation might decrease lung elastance (intra-tidal recruitment) [40] or resistances (inversely correlated with tidal volume [41]), thereby partially re-establishing the matching between the brain and the ventilation curve. On the other hand, neuromechanical uncoupling may worsen if PEEP is increased without corresponding recruitment [42] or if muscular fatigue is associated with inadequate support. Accordingly, the dissociation between the brain and the ventilation curve may increase (see Panel D in Fig. 2).

There are other important interactions which are particularly relevant in the spontaneously breathing patient:

1. With increasing VCO_2NL_, shifting the metabolic hyperbola upward and to the right would result in increased PaCO_2_ if the brain curve did not concomitantly change position (see Fig. 2 Panel B). However, it has been experimentally shown that PaCO_2_ remains constant at decreasing SGF, unless extreme effort is reached [20]. A similar behaviour of the respiratory centres occurs in exercise, where increasing VCO_2NL_ is associated with a parallel leftward shift of the brain curve (at constant slope) to maintain constant PaCO_2_ (isocapnic hyperpnea) [37]. The underlying mechanism explaining this phenomenon remains debated [43].
2. When the brain curve shifts to lower PaCO_2_ set points, the entire pool of CO_2_ body stores must equilibrate with the new PaCO_2_ (see Fig. 2 panel C). This requires displacement of a vast amount of non-metabolic CO_2_ from peripheral tissues into the bloodstream increasing V’CO_2NL_ [44] and further shifting the metabolic hyperbola upward and to the right. The higher the pool of total body CO_2_ stores (for example due to prior permissive hypercapnia), the greater the amount of CO_2_ displaced to reach equilibrium and therefore the greater V’CO_2NL_ which may be required to maintain the new set-point.
3. The slope of the metabolic hyperbola, describing how much VE must change to obtain a given change in PaCO_2_ (the so-called “plant gain”) has two characteristics which are relevant to weaning. First, it is lower at higher VE [38] (see Fig. 2 panel A). Therefore, during weaning, higher changes in VE are required to achieve a new PaCO_2_ set-point if the patient is already hyperventilating prior to the trial. This might be one reason why high breathing effort before or during a weaning trial has been associated with weaning failure [19, 20]. Second the slope decreases when the V’CO_2ML_ is decreased [37]. Accordingly reaching a new PaCO_2_ set-point (e.g. because of hypoxaemia) requires much more effort during the later stages of a weaning trial. For both reasons, avoiding hypoxaemia, distress or any other cause for a shift in the eupneic threshold is important during a weaning trial.

#### Monitoring respiratory centres output

Respiratory centres can express their output in terms of timing or intensity: the timing is reflected by respiratory rate, while the intensity of output is referred to as respiratory drive. Respiratory rate significantly increases only when respiratory drive is 3–4 times elevated [35, 36]. Similarly, clinical signs of high effort occur when drive is already excessive. For this reason, invasive assessment of drive is necessary to predict the success or failure of weaning prior to the development of overt distress. Directly measuring the rate of change of the electrical activity of the brain centres is not feasible in routine practice, thereby surrogates need to be employed. These surrogates relate more or less directly to respiratory drive (the electrical activity of the diaphragm, EAdi [45]), others with respiratory effort (P0.1 [46], the swing in oesophageal, ∆P_eso_, and transdiaphragmatic pressure, ∆P_di_ [47], or the muscle pressure, P_musc_, and the occlusion pressure, P_occ_ [48]), others with lung stress (dynamic transpulmonary pressure, ∆PL [47, 48]). If the descending pathway is altered (neuromuscular impairment, increased respiratory system elastance), as typically seen in patient undergoing V–V ECMO, a dissociation between these indices might occur, thereby complicating the assessment of the respiratory centres output.

### The ventilator

We have discussed the effects of weaning extracorporeal parameters on V’O_2ML_ and V’CO_2ML_ together with the resulting interactions with the patient’s respiratory centres. In the following section, we will discuss the approach to mechanical ventilation during a weaning trial.

#### Passive controlled patients

In fully sedated patients in controlled modes, changes in extracorporeal gas exchange may be directly reflected in the systemic arterial blood gases if concomitant changes in mechanical ventilation are not made. For safe decannulation from V–V ECMO, maintenance of gas exchange must not be at the cost of excessive risk of ventilator induced lung injury (VILI). Overall, protective ventilation during V–V ECMO is debated [49]. The holistic concept of mechanical power [50] particularly highlights the harms of respiratory rate as well as driving pressure [51], though safety thresholds are unclear [52, 53]. Associations between mortality and the use of higher driving pressure [54], and mechanical power [55] during V–V ECMO have been made from cohort studies and a period of total lung rest with zero driving pressure was correlated with lower plasma biomarkers of lung injury in a recent small randomized trial [56]. However, ultra-protective ventilation may be unnecessarily cautious when patients have improved to the point of a weaning trial off. Finally, increased sedation or even neuromuscular blockade may be required to maintain synchrony if ventilation is not adequately adjusted to match the demands of the brain curve.

#### Spontaneously breathing patients

The spontaneously breathing patient’s respiratory centre’s output will dynamically increment to maintain gas exchange as extracorporeal support is weaned. Changes in respiratory centres output during weaning translate into breathing effort and lung stress which is the main contributor to patient self-induced lung injury (P-SILI) [57]. The role of the ventilator in reducing breathing effort and stress is crucial and can be divided into two components:

#### Manoeuvres reducing effort and stress

Any manoeuvre shifting the CO_2_ sensitivity curve to higher PaCO_2_ or improving the matching between the ventilation and the brain curve may reduce breathing effort and lung stress [35, 36]. The use of sedation, shifting the CO_2_ sensitivity curve to the right, is a typical ‘non-ventilatory’ strategy in this regard. Increasing FiO_2_ and PEEP (if associated with recruitment) may have beneficial effects. However, increasing PEEP may worsen neuromechanical uncoupling [42] and it can increase the *static* stress to the lung contributing to the total mechanical power of ventilation [50, 58]. Manoeuvres improving patient–ventilator synchrony also have the potential to reduce effort and regional stress [57].

#### Manoeuvres reducing effort but not stress

Any manoeuvre unloading the respiratory muscles (e.g. increasing pressure support) has the potential to reduce breathing effort, but not lung stress. Indeed, when the respiratory centres are set to maintain a certain PaCO_2_, pressure support leads to a decreased workload to the respiratory muscles, but the total ventilation required to maintain PaCO_2_ does not change (i.e. stress will not decrease) [59].

Irrespective of the manoeuvre performed, general principles of lung protection during a weaning trial in spontaneously breathing patients are similar to those in fully sedated patients under controlled ventilation (Table 4 [14, 19, 20, 57] summarizes possible stopping criteria of a weaning trial from V–V ECMO). However, some important specifics must be highlighted:

1. The presence of active muscular contraction, variable between inspiration and expiration, confounds the estimation of chest wall compliance. One implication is that whole respiratory system indices like driving pressure might less reliably reflect lung stress than during passive ventilation. Additionally, abdominal muscle contraction may both reduce the static stress associated with PEEP [60, 61] and alter the estimation of effort with oesophageal pressure if gastric pressure is not concomitantly measured [47].
2. Indices of effort represent an “average” measurement of the stress applied to the lung. Indeed, in spontaneous breathing, significant regional changes in oesophageal and transpulmonary pressure can occur [62]. This may elicit pendelluft and negative pressure alveolar oedema, worsening P-SILI [57]. Although PEEP can increase lung homogeneity counteracting these effects [63, 64], its downsides must be kept in mind.

**Table 4:** Stopping criteria during weaning from V–V ECMO

Parameter	Values of concern	Downsides
Oxygen saturation	< 88%	Late sign of distress
Heart rate	> 110	Multifactorial causes
PaCO_2_	New respiratory acidosis	Late sign of distress
Respiratory rate	> 35	Late sign of distress
Tidal volume	> 8 ml/kg IBW	Depends on respiratory system elastance
Driving pressure	> 15 cm H_2_O	Evaluates both lung and chest wall
P 0.1	> 10 cmH_2_O	May be falsely low in patients with respiratory muscle weakness
∆P_eso_	< − 15 cmH_2_O	Requires an oesophageal catheter
P_occ_	< − 20 cmH_2_O	Requires multiple manual manoeuvres
P_musc_*	> 10 cmH_2_O	Requires measurement (or estimation) of chest wall elastance
∆P_L_	> 20 cmH_2_O	Requires oesophageal catheter
Total lung stress (PEEP_L_ + ∆P_L_)	Unknown	Difficult assessment of PEEP_L_ in patients with abdominal contraction

PEEP_L_: static stress associated with PEEP

*P_musc_ can be derived from the oesophageal pressure swing and the estimated chest wall elastance. Alternatively, it can be estimated from − 0.75 × P_occ_

**Fig. 3:**
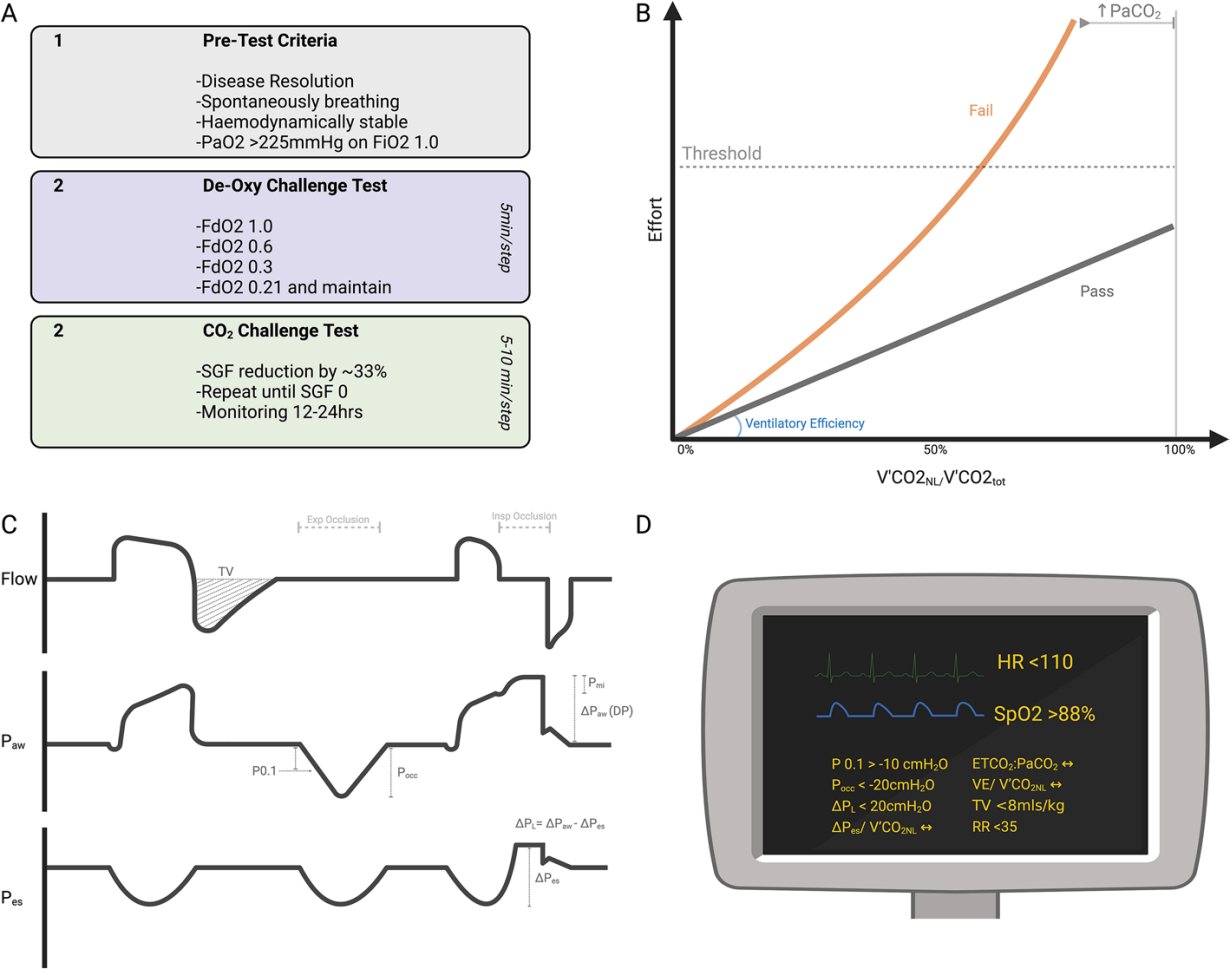
Our process of weaning V–V ECMO. **A** Sequence for V–V ECMO weaning. Throughout, monitor for stopping criteria. **B** As SGF is weaned, the proportion of metabolically produced CO_2_ cleared by the natural lung (V’CO_2_NL/V’CO_2_tot) increases. In patients who are not yet suitable for decannulation, this load can only be managed with excessive effort (see next panels). Ventilatory efficiency can be expressed as the ratio of the effort (or minute ventilation) to the V’CO_2NL_. In patients who fail a weaning trial ventilatory efficiency is usually worse, and may deteriorate as the demands on the respiratory system rise. If they are not able to clear all of the metabolically produced CO_2_ then hypercapnia ensues. **C** Monitoring drive and effort during a weaning trial. Waveforms during a pressure supported breath, an expiratory occlusion throughout an inspiratory cycle and an end inspiratory occlusion. **D** Targets to maintain during a trial off V–V ECMO including measures of drive (P0.1), effort (Pocc or ∆P_es_ if available), stress (∆P_L_ or DP if not available) and native lung ventilator efficiency (∆P_es_/V’CO_2NL_, end tidal CO_2_ to arterial CO_2_ ratio (ETCO_2_:PaCO_2_) or the ratio of minute ventilation to clearance (VE/ V’CO_2NL_). At our centre, volumetric capnography from the ventilator and pre- and post- oxygenator blood gases are used to calculate the V’CO_2NL_ and V’CO_2ML_, respectively. Unfortunately, during weaning the V’O_2NL_ is not routinely measured. However, this is done in individual patients who have a pulmonary artery catheter for measurement of C_Vmixed_O_2_ or a receive calorimetric measurements. FdO_2_: the fraction of oxygen of the sweep gas flow (SGF), TV: tidal volume, P0.1: pressure deflection during 100 ms of occlusion, Pocc: maximal pressure deflection during occlusion, ∆P_es_ oesophageal pressure swing, ∆P_aw_: plateau after inspiratory inclusion, including the PMI: rebound pressure from relaxing inspiratory muscles, ∆P_L_: transpulmonary pressure

Importantly, if the patient is spontaneously breathing, the development of hypercapnia or respiratory distress are late signs (panel B Fig. 3). Accordingly, measurement of drive and effort is essential to optimize mechanical ventilation and avoid exposing the patient to P-SILI and premature decannulation. Even with ‘protective’ parameters measured from the airway, the spontaneous effort to maintain normocapnia can generate enormous transpulmonary pressures [65]. Increased tidal volumes (driven by increased dead space) and new tachycardia predicted unsafe decannulation in a recent case series, but ∆P_es_ of > 16 cmH_2_O had the greatest accuracy [20].

## A proposed approach to weaning

Our approach to weaning is represented in Fig. 3 [14]. Prior to commencing weaning comprehensive assessment of respiratory drive, effort, mechanical ventilation and the CO_2_ clearance capacity of the lung should take place, both to optimize mechanical assistance and as a baseline measure. The V’CO_2NL_ should be at least 50% of the total metabolically produced carbon dioxide. Our preference is for patients to be on a spontaneous or assisted mode of ventilation. First the FiO_2_ is set to 0.6 in anticipation of reducing V’O_2ML_ and to avoid alveolar hypoxia as the respiratory quotient is changed during weaning. The ECBF is held static unless there is suspicion of an intracardiac shunt. Next, the FdO_2_ is sequentially weaned in 5-min intervals, allowing for re-establishment of hypoxic pulmonary vasoconstriction and potentially increasing native ventilatory efficiency with improved V/Q matching. If an FdO_2_ of 0.21 is tolerated, next the SGF is sequentially decreased to zero in 5–10 min intervals. As the V’CO_2ML_ falls, monitoring continues to ensure increasing V’CO_2NL_ is not at the cost of injurious effort or stress. In all patients, the total V’CO_2_ will rise due to the increasing work of breathing to manage the load from the V’CO_2ML_. Depending on the pulmonary mechanics and ventilatory efficiency, in some patients the native lungs will not be capable of managing this load and they will demonstrate increasing respiratory drive, effort, minute ventilation, sympathetic activation and ultimately hypercapnia. Time to equilibrate at each step is essential in order to fully assess response and prevent P-SILI. Throughout, multimodal monitoring is continued, and the weaning test is ceased if there are indices of concern (see Table 3 and Fig. 3). As decannulation itself often produces a systemic inflammatory response in the following days, our preference is for a conservative approach to decannulation and a trial off SGF of 24 h.

## Conclusions

The rationale for the use of V–V ECMO in ARDS and other forms of severe respiratory failure is becoming clearer. However, variation in mortality between centres [8] and a lack of prospective randomized evidence regarding the management of patients on V–V ECMO means there is a strong scientific rationale for further study. Early, safe liberation from V–V ECMO has the potential to hasten patient’s recovery and maintain equity of access to other patients who may benefit from this effective, but resource-intensive treatment. Although there is not yet high-grade evidence to guide clinicians, we have outlined an approach to weaning underpinned by physiology. The feasibility of weaning from V–V ECMO should be considered daily. We advocate separating the ability of the natural lung to provide O_2_ and remove CO_2_ by weaning the FdO_2_ prior to the SGF. The complex interactions between the determinants of respiratory drive, the patient’s effort and ventilatory assistance, their lung mechanics and efficiency of V’CO_2NL_ will determine the outcome of a weaning trial. Care must be taken to avoid occult P-SILI in patients making spontaneous effort and we advocate multimodal assessment of drive, effort and stress throughout the weaning process.

## Data Availability

Not applicable to this narrative review.
